# Lowered Diversity and Increased Inbreeding Depression within Peripheral Populations of Wild Rice *Oryza rufipogon*

**DOI:** 10.1371/journal.pone.0150468

**Published:** 2016-03-10

**Authors:** Li-Zhi Gao, Cheng-Wen Gao

**Affiliations:** 1 Plant Germplasm and Genomics Center, Germplasm Bank of Wild Species in Southwest China, Kunming Institute of Botany, The Chinese Academy of Sciences, Kunming, 650204, P. R. China; 2 Faculty of Life Science and Technology, Kunming University of Science and Technology, Kunming, 650504, China; Meiji University, JAPAN

## Abstract

**Background:**

The distribution of genetic variability from the interior towards the periphery of a species’ range is of great interest to evolutionary biologists. Although it has been long presumed that population genetic variation should decrease as a species’ range is approached, results of empirical investigations still remain ambiguous. Knowledge regarding patterns of genetic variability as well as affected factors is particularly not conclusive in plants.

**Methodology/Principal Findings:**

To determine genetic divergence in peripheral populations of the wild rice *Oryza rufipogon* Griff. from China, genetic diversity and population structure were studied in five northern & northeastern peripheral and 16 central populations using six microsatellite loci. We found that populations resided at peripheries of the species possessed markedly decreased microsatellite diversity than those located in its center. Population size was observed to be positively correlated with microsatellite diversity. Moreover, there are significantly positive correlations between levels of microsatellite diversity and distances from the northern and northeastern periphery of this species. To investigate genetic structure and heterozygosity variation between generations of *O*. *rufipogon*, a total of 2382 progeny seeds from 186 maternal families were further assayed from three peripheral and central populations, respectively. Peripheral populations exhibited significantly lower levels of heterozygosities than central populations for both seed and maternal generations. In comparisons with maternal samples, significantly low observed heterozygosity (*H*_*O*_) and high heterozygote deficit within populations (*F*_*IS*_) values were detected in seed samples from both peripheral and central populations. Significantly lower observed heterozygosity (*H*_*O*_) and higher *F*_*IS*_ values were further observed in peripheral populations than those in central populations for seed samples. The results indicate an excess of homozygotes and thus high inbreeding depression in peripheral populations.

**Conclusions/Significance:**

Our results together suggest that historical contraction of geographical range, demographic changes, and environmental conditions near the northern and northeastern margins of *O*. *rufipogon* favor inbreeding and possibly selfing, leading to the rapidly decreased effective population size. Genetic drift, reduced gene flow, and possible local selection, consequently lead to lowered gene diversity, accelerated genetic divergence and increased inbreeding depression found in peripheral populations of *O*. *rufipogon*. Given these characteristics observed, northern and northeastern peripheral populations deserve relatively different conservation strategies for either germplasm sampling of *ex situ* conservation or setting *in situ* reserves for the adaptation to possible environmental changes and the future germplasm utilization of wild rice.

## Introduction

The partitioning of genetic variability on both local and large geographical scales, in either animal or plant species, is of great interest to ecologists and conservation biologists. As one moves from the interior towards the periphery of a species’ range, the spatial distribution, dynamics and structure of populations change [1]. Populations often become more patchy, isolated and transient, and their probability of extinction increases towards the edge of the range [2–4]. Consequently, population genetic theories suggest that peripheral populations will diverge from central populations as a result of two important processes: genetic drift and natural selection [1, 5]. Peripheral populations of a species, which exist at the ecological periphery of the species range, usually have low population density due to unfavorable ecological conditions, and thus reduce the possibility of the persistence of individual populations [6, 7]. However, recent studies challenge the above-mentioned commonly recognized viewpoint that peripheral populations are demographically reduced and are likely to suffer local extinction events. For instance, Channell & Lomolino [8] found that peripheral populations experience fewer extirpations than centrally located populations because of range contractions that are not predicted from historical distributions and immigration rates. With this regard, one should not ignore the fact that the present-day periphery has undergone extinction and reduction as a result of the historical contraction of the geographical range of a species. The historical consequences of population demography may have played an important role in the observed difference of genetic structure in peripheral populations. In addition, novel alleles proven in peripheral populations may potentially help their adaptation and evolution. Therefore, to further understand how genetic variation in nature is partitioned among peripheral and central populations would contribute much to our knowledge of the evolution in peripheral populations.

Although it has been long presumed that population genetic variation should decrease as a species’ range is approached, results of empirical investigations still remain ambiguous. In addition to the increasing references in animal studies [9–11], numerous studies of plants have compared population genetic diversity between central and peripheral populations by using morphological, allozyme, RAPDs, ISSR, and microsatellite analyses. Peripheral populations exhibited reduced genetic variation in comparison with central populations [12–17]. However, investigation of peripheral populations in several plant species did not show reduced genetic variation compared to central populations [18–20]. In contrast, some researchers reported that peripheral populations exhibited even larger amounts of genetic diversity than those located in the centers of the species range [21–23]. Therefore, knowledge regarding patterns of genetic variability as well as affected factors is not conclusive in plants.

The common wild rice, *Oryza rufipogon* Griff., is a perennial herbaceous species with a mixed mating system, primarily growing on ditches, ponds, and swamps, or along rivers, streams, and lakes. It is widely distributed in the tropics and subtropics of monsoon Asia [23–25]. As extremely important gene sources for the breakthrough of the world rice breeding program [26], the species has been extensively explored and collected throughout the world, and thus its geographical range has been well documented to date [25]. Field investigation suggested that this species has a distribution centered in China; all the northern and northeastern peripheral populations of the species were clearly identified within this region [27]. Worldwide evaluation of genetic diversity in *O*. *rufipogon* suggested that the Chinese populations possessed higher levels of genetic diversity than those from other geographical origins [28]. In addition, the species has been under considerable threats in China [29], of which peripheral populations have been seriously endangered and have experienced rapid genetic erosion [30]. Peripheral populations of the wild rice are important components of the whole rice gene pool because they have been proven to find novel abiotic alleles (e.g., cold tolerance genes) [31]. With this regard, there is no doubt concerning the importance of peripheral populations of this wild rice for the continuous evolution and its value for conservation and breeding programs. A comparison of levels and partitioning of genetic diversity within its peripheral and central populations would undoubtedly benefit conservation management of genetic resources of wild rice as well as further rice germplasm utilization.

In this context, the aim of the present study was to investigate the population structure and genetic diversity of the northern and northeastern peripheral populations of *O*. *rufipogon*. Using microsatellite analysis, five peripheral populations were examined, and in comparison, 16 more centrally located populations were selected. We are specifically interested in the following questions: 1) Do the populations located on the periphery of the species range have reduced genetic variability with respect to more centrally located populations? 2) Do peripheral populations have a significant genetic structure that is different from those in the center? 3) Is the genetic diversity of this species influenced by population size? 4) Does inbreeding depression affect genetic structure and make difference between peripheral populations and those located in the center? and 5) What are conservation implications based on the observed patterns of genetic variability in the wild rice species?

## Materials and Methods

### Study area and populations

**Fig 1:**
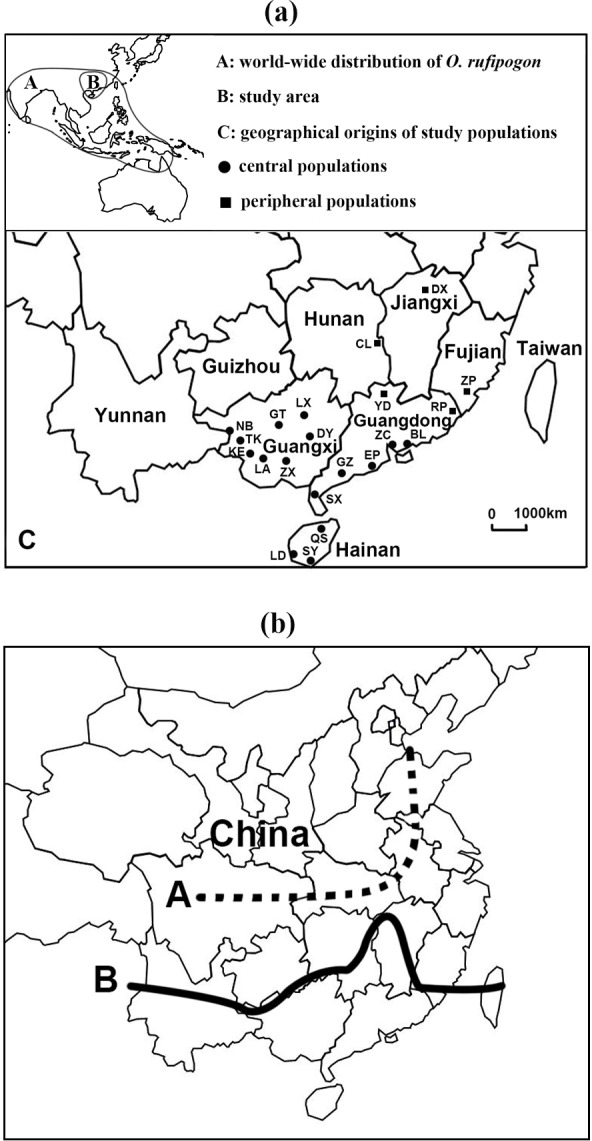
World-wide distribution and geographical origins of the study populations of *O*. *rufipogon* (a), and comparison of the past (A) and current (B) range of *O*. *rufipogon* in China (b).

*O*. *rufipogon* mainly grows in southern China with great abundance in large populations (Fig 1). For example, in Guangdong and Hainan provinces, a total of 1,182 populations were historically documented, most of which were large in population size [32]. This region has a structurally rich mosaic of diverse habitat types, together with favorable ecological conditions for the growth of the *O*. *rufipogon* populations, hot and humid weather in the typical tropics and subtropics. However, the species becomes rather small in isolated patches while extending to its northern (28° 14’ N, 116° 36’ E) and northeastern (24° 10’ N, 117° 08’ E) ranges. For the purpose of sampling populations, it is important to define the periphery and core of the species. Most workers estimated the periphery, or consider peripheral only those populations remotely isolated at the geographical extent of a species’ range. However, there were several studies that gave definitions of periphery [8, 10, 33]. For example, Channell & Lomolino [8] defined the periphery as the region that is within half the distance to the edge of a species’ geographical range from a central point. In this study, we adopted the above-mentioned definition [8] to categorize the study populations as peripheral or central as located in Fig 1. The southernmost population (SY) of China was set as the central point of the species geographical range. *O*. *rufipogon* occurs in Australia, Bangladesh, China, India, Indonesia, Laos, Malaysia, Myanmar, Nepal, Papua New Guinea, Philippines, Sri Lanka, Thailand, and Vietnam [25] (Fig 1). Therefore, the location used in this study may not accurately represent the central point of the whole geographical range. However, for such a plant species with a disjunct distribution, it seems reasonable to use the southernmost population of the study region as a central point to define its periphery since China holds northern and northeastern regions of the whole species range. According to the above-mentioned definition, a total of five populations (RP, ZP, YD, CL, DX) fell into the periphery (peripheral populations) while other 16 populations corresponded to the core (central populations). In this study, all northern and northeastern populations were sampled, although peripheral populations still seem unevenly fewer than central populations. Geographical locations of these twenty-one study populations were shown in Fig 1. Detailed information regarding population size, habitat types, and geographical locations were also given in Table 1.

**Table 1:** Population codes, geographical origins and sample sizes of the 21 studied populations of *O*. *rufipogon*.

Population codes	Geographical origins	Latitude (°N)	Longitude (°E)	Sample sizes	Population sizes^†^	Habitat types	Ecological designation
**DY**	Guigang, Guangxi	23.18	109.73	27	6,000	Ponds	Central
**LX**	Wuxuan, Guangxi	23.53	109.54	25	5,000	Streams	Central
**GT**	Bingyang, Guangxi	22.98	108.99	27	2,000	Ponds, ditches	Central
**NB**	Beise, Guangxi	24.10	105.82	24	1,500	Ponds, ditches	Central
**ZX**	Nanning, Guangxi	22.77	108.09	24	550	Ponds, ditches	Central
**LA**	Longan, Guangxi	23.04	107.87	25	1,000	Ponds, ditches	Central
**TK**	Tiandong, Guangxi	23.59	107.12	18	1,500	Ponds, ditches	Central
**KE**	Tiandong, Guangxi	23.55	107.16	25	3,000	Ponds, ditches	Central
**QS**	Qiongshan, Hainan	19.97	110.39	28	2,500	Ponds, ditches, streams	Central
**SY**	Sanya, Hainan	18.36	109.17	24	300	Ponds	Central
**LD**	Ledong, Hainan	18.47	108.85	28	3,000	Ponds, ditches, streams	Central
**SX**	Suixi, Guangdong	21.38	110.25	30	2,000	Ponds, streams	Central
**EP**	Enpin, Guangdong	22.27	112.22	29	10,000	Ponds, streams	Central
**ZC**	Zengcheng, Guangdong	23.41	113.77	28	5,000	Marshes, rivers	Central
**BL**	Boluo, Guangdong	23.45	114.44	26	2,000	Streams	Central
**GZ**	Gaozhou, Guangdong	21.86	110.70	27	10,000	Ponds, ditches, marshes	Central
**RP**	Raoping, Guangdong	23.66	116.98	31	500	Ponds	Peripheral
**YD**	Yingde, Guangdong	24.25	113.13	30	150	Lotus ponds	Peripheral
**DX**	Dongxiang, Jiangxi	28.34	116.71	29	120	Ponds	Peripheral
**ZP**	Zhangpu, Fujian	24.18	117.83	25	150	Ponds, ditches	Peripheral
**CL**	Chaning, Hunan	26.72	113.49	25	180	Pond	Peripheral

^†^ Population size is the estimation of number of flowering plants per population.

### Plant material sampling

Dry leaves of a total of 525 individual plants from 21 populations representing peripheral and central populations of *O*. *rufipogon* in China were collected between November and December of 2000 (Table 1). All necessary permits were obtained from Xu Liu, who was Vice President, the Chinese Academy of Agricultural Sciences. Young, unblemished leaves were individually collected from at least 20 plants per population. Because *O*. *rufipogon* is a spreading perennial herb with clonal growth, samples were randomly collected at an interval of at least 5 meters to prevent the collection of multiple samples from a single genet. Leaves were silica-dried following the method described by Chase & Hills [34]. For each study population, geographical distances from the northern and northeastern periphery (DX and ZP, respectively) were estimated and recorded based on their geographical locations in the map.

To study genetic structure and heterozygosity variation between generations of *O*. *rufipogon*, both seeds and maternal plants were further collected from three peripheral (DX, CL and RP) and central (GZ, LD and LX) populations from November to December of 2000. More than 25 individuals were randomly sampled in each population. All the seeds were collected from respective maternal plants, and all the seeds from one maternal plant were referred to as a family. For these six natural populations, a total of 2,382 seeds from 186 families were assayed.

### Microsatellite screening

Total DNA from the silica-dried leaves of a single individual or young leaves from a single germinated seed was isolated according to the protocol of Edwards *et al*. [35]. Six microsatellite primer pairs (RM164, RM241, RM211, RM253, OSR28, RM222) were used, which were developed in cultivated rice *O*. *sativa* [36–39]. These six polymorphic SSR loci were selected in a total of 493 publicly available SSR markers by screening 30 individuals from geographic representative populations based on their allele richness. Detailed information of these primer sequences is now available at http://www.gramene.org/microsat/ssr.txt. Microsatellite polymorphisms were analyzed using specific polymerase chain reaction (PCR) conditions as described in Panaud *et al*. [38]. Microsatellite fragments were resolved by 4% polyacrylamide denaturing gels. The gels were stained with the silver staining as described by Panaud *et al*. [38]. To determine allele size, samples were directly compared with band sizes from an allelic ladder, which were prepared by amplification of an artificial mixture of DNA from the twenty-one *O*. *rufipogon* populations.

### Data analyses

Genetic parameters overall and for each population were assessed by calculating the allelic richness (*Rs*), the observed heterozygosity (*H*_*O*_), the gene diversity within the sample (*H*_*S*_), and the heterozygote deficit within populations (*F*_*IS*_). To avoid the lower sample sizes in the peripheral group could lead to a lower detection probability of genetic variation, allelic richness for specified sample sizes was computed with the rarefaction method developed by Hurlbert [40] using the software HP-rare 1.0 [41]. In this study, five sample populations (the total sample size of peripheral populations) were used as rarefaction sample sizes from each belonged group (central or peripheral). Moreover, we applied AMOVA using the program Arlequin 1.1 [42] to assessing the partioning of the genetic variance within and among populations, and among central and peripheral groups of populations; for each analysis, 16, 000 permutations were computed in order to obtain the significance levels of the variance.

Overall estimates for two groups of populations were also obtained by pooling central and peripheral populations, respectively. *H*_*O*_ and *F*_*IS*_ were calculated using GENEPOP version 3.1c [43] while *Rs* and *H*_*S*_ were evaluated by FSTAT version 2.9.3 [44]. To quantify population genetic differentiation, Weir & Cockerham [45] estimators of *F*_*IT*_, *F*_*ST*_ and *F*_*IS*_ were estimated for each locus and overall by pooling peripheral and central populations, respectively, using FSTAT version 2.9.3 [44]. Bootstrapping over loci was automatically performed for the statistics. Tests for the presence of population differentiation were also made by using an unbiased estimated *P*-value for a log-likelihood (*G*)-based exact test [46] with FSTAT version 2.9.3 [44]. A Mantel test [47] was performed between the two matrices of genetic differentiation and geographic distances to test for a pattern of isolation by distance. Outcrossing rate was roughly estimated by *t* = (1- *F*_*IS*_)/(1+ *F*_*IS*_) [48]. To examine whether gene flow is responsible for the reduction of genetic diversity in the study populations from the periphery of the species, regression tests were further performed between levels of genetic diversity and distances of the population from northern and northeastern periphery. Significant differences in genetic structure parameters either between seed and maternal populations or peripheral and central populations were examined by the *t* test [49].

## Results

### Genetic variability within peripheral and central populations

In this study, genetic variability in a total of the 21 natural populations was estimated using the six microsatellite loci. Microsatellite loci (mean values of *Rs* = 3.4331, *H*_*O*_ = 0.2281, and *Hs* = 0.6766), as expected, detected much higher levels of genetic diversity for the populations studied here (Table 2) than allozyme loci (*A* = 1.33, *P* = 22.7%, *H*_*E*_ = 0.068) reported previously [50]. While pooling central and peripheral populations, respectively, average estimates of genetic diversity for the central population group (*Rs* = 3.518, *H*_*O*_ = 0.217, and *Hs* = 0.664) were significantly larger than those for the peripheral population group. In addition, estimates of allelic richness at three of six loci significantly higher for the central populations than those for the peripheral populations with the rarefaction method (*P* < 0.05) (Table 3). One northern peripheral population (YD) from Guangdong Province showed markedly larger genetic diversity than many of the detected populations from the center (e.g., DY, GT, NB, SY, SX, EP, BL). Of these studied populations, the northernmost population (DX) harbored the lowest genetic diversity.

**Table 2:** Genetic variation in central and peripheral populations of *O*. *rufipogon* across six microsatellite loci ^a^.

Populations	*Rs*	*H*_*O*_ ^b^	*H*_*S*_	*F*_*IS*_ ^b^	*t*
**DY**	3.285	0.2292 (0.1363) ***	0.5770	0.603***	0.248
**LX**	3.904	0.2549 (0.1810) ***	0.7472	0.659***	0.206
**GT**	3.230	0.2444 (0.0807) ***	0.6790	0.640***	0.220
**NB**	3.109	0.2222 (0.1721) ***	0.6471	0.657***	0.207
**ZX**	4.133	0.3056 (0.2722) ***	0.8060	0.618***	0.236
**LA**	3.671	0.5000 (0.2449)	0.7042	0.290**	0.550
**TK**	3.000	0.3333 (0.2041)	0.7222	0.538***	0.300
**KE**	4.001	0.5500 (0.2429) ***	0.7683	0.284***	0.558
**QS**	4.162	0.1933 (0.1275)***	0.7793	0.749***	0.144
**SY**	3.075	0.1053 (0.0815)***	0.6498	0.837***	0.089
**LD**	3.739	0.1600 (0.0912)***	0.7345	0.781***	0.123
**SX**	3.414	0.1933 (0.1628)***	0.6630	0.703***	0.174
**EP**	3.394	0.2431 (0.1997)***	0.6617	0.633***	0.225
**ZC**	3.842	0.1597 (0.0965)***	0.7390	0.784***	0.121
**BL**	3.660	0.1852 (0.0907)***	0.7002	0.725***	0.159
**GZ**	4.457	0.2419 (0.0860)***	0.8040	0.699***	0.177
**Pooling central populations**	**3.518 (0.436)** ^d^	**0.2170 (0.1185)**	**0.6640 (0.0633)**	**0.673 (0.157)**	**0.195 (0.136)**
**RP**	2.501	0.1389 (0.1394)***	0.4870	0.715***	0.166
**YD**	3.641	0.2917 (0.1021)***	0.7170	0.592***	0.256
**DX**	2.389	0.0556 (0.0557)***	0.4905	0.884***	0.062
**ZP**	2.941	0.1078 (0.0443)***	0.6271	0.826***	0.095
**CL**	2.548	0.0750 (0.0524)***	0.5046	0.844***	0.085
**Pooling peripheral populations**	**2.776 (0.512)**	**0.1360 (0.0938)**	**0.5560 (0.1028)**	**0.755 (0.119)**	**0.140 (0.079)**
***P*-value** ^c^	0.0021	0.0471	0.0010	0.0946	0.136
**Total**	**3.433 (0.570)**	**0.2281 (0.1234)**	**0.6766 (0.0958)**	**0.670 (0.157)**	**0.198 (0.131)**

^a^ Allelic richness (*Rs*), the observed heterozygosity (*H*_*O*_), the gene diversity within sample (*H*_*S*_), the heterozygote deficit within populations (*F*_*IS*_), and the estimate of outcrossing rate (*t*) for each studied population of *O*. *rufipogon* across six loci; mean values (*Rs*, *H*_*O*_, *H*_*S*_, *F*_*IS*_ and *t*) are also given by pooling all central and peripheral populations, respectively.

^b^ Statistically significant deviations from Hardy-Weinberg expectations are indicated by

** (*P* < 0.01) and

*** (*P*<0.001).

^c^ The two-sided *P*-values obtained after 1,000 permutations.

^d^ The standard deviations of the means are given in the parenthesis.

**Table 3:** Allelic richness in central and peripheral populations of *O*. *rufipogon* across six microsatellite loci using rarefaction method.

Populations	RM164	RM241	RM211	RM253	OSR28	RM222
**DY**	4.7694	2.0553	2.1624	2.3744	4.5109	3.8364
**LX**	2.5553	5.9008	3.2696	4.3207	4.5606	2.8149
**GT**	1.9162	4.6987	2.9308	2.7248	4.222	2.8885
**NB**	3.7537	3.1172	2.839	2.9475	2.8854	3.1123
**ZX**	3.4274	4.2898	4.6637	3.7708	4.4287	4.2154
**LA**	4.2	5.5111	2.9556	2	4.5778	2.7778
**TK**	3	4	4	3	2	2
**KE**	3.2031	4.7154	3.5465	4.5282	4.4907	3.5227
**QS**	1	4.873	4.5164	4.6201	4.5986	3.7858
**SY**	1	3.0645	2.7604	2.9238	3.9584	3.4476
**LD**	1	3.3897	4.6303	3.3028	4.4975	3.5069
**SX**	1	2.7524	4.2702	3.5764	4.4311	2.5978
**EP**	4.6347	3.8098	2.7478	2.4007	3.4823	3.2891
**ZC**	1	3.6937	4.8994	3.3573	4.4002	4.1165
**BL**	2.6366	4.1116	4.9465	3.0812	4.5461	2.6366
**GZ**	1	4.9039	5.3208	4.6287	4.4305	4.202
**Pooling central populations**	**8.4945 (1.4056)**^a^	**13.2584 (1.0334)**	**9.3676 (0.9881)**	**8.9334 (0.8332)**	**10.319 (0.7337)**	**8.9892 (0.6477)**
**RP**	1.8582	1.6061	1.9615	2.8867	3.8505	2.8442
**YD**	4.306	3.5977	2.9769	3.4747	4.8112	2.6783
**DX**	1	1.9711	2.0213	3.063	2.5191	2.9108
**ZP**	3.3394	2.8915	2.3801	3.2964	2.9724	2.7689
**CL**	1.9921	2.8428	1.7641	4.2269	2.5045	1.9596
**Pooling peripheral populations**	**6.6569 (1.3121)**	**7.2244 (0.7939)**	**5.1908 (0.4777)**	**6.3587 (0.5188)**	**8.0883 (0.9914)**	**4.8958 (0.3859)**
***P*-value**	0.9924	0.0094	0.0036	0.9168	0.0664	0.0448
**Overall**	**11.8834 (1.3513)**	**15.3222 (1.1578)**	**9.74 (1.1137)**	**9.4359 (0.7582)**	**11.5196 (0.8489)**	**9.7152 (0.6546)**

^a^ The standard deviations of the means are given in the parenthesis.

### Population size, geographical distance and genetic diversity within peripheral and central populations

**Fig 2:**
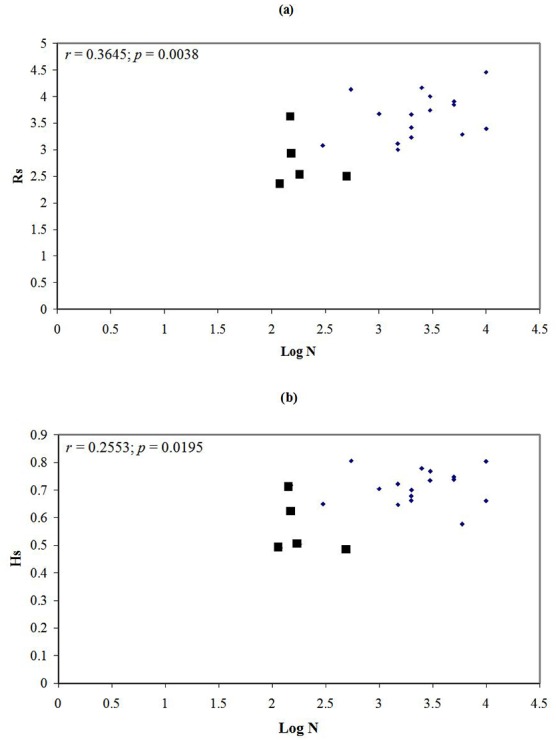
**Relationship between logarithm of population size and microsatellite diversity: allelic richness (a), and gene diversity (b).** solid squares indicate the peripheral populations, while others are central populations.

In this study, Pearson Coefficients were used to estimate the correlations between population size, geographical distance and genetic diversity within peripheral and central populations, respectively. Population size was positively correlated with microsatellite diversity (*n* = 21; *Rs*: *r* = 0.3645, *P* = 0.0038; *Hs*: *r* = 0.2553, *P* = 0.0195) (Fig 2). Our data also showed significant correlations between levels of genetic diversity and distances of populations from the northern margin (DX, CL, YD) (*Rs*: *r* = 0.2791, *P* = 0.0138; *Hs*: *r* = 0.3822, *P* = 0.0028) and from the northeastern margin (ZP, RP) (*Ho*: *r* = 0.2435, *P* = 0.0230; *Hs*: *r* = 0.1897, *P* = 0.0485).

### Genetic structure of peripheral and central populations

Genetic variation detected in peripheral and central populations was portioned into within- and between- population components by *F*-statistics. The *F*_*ST*_ estimate among peripheral populations (*F*_*ST*_ = 0.266) was slightly higher than that among central populations (*F*_*ST*_ = 0.233), but the difference was not significant (*P* = 0.6461) (Table 4). The *F*_*ST*_ estimates did not significantly correlate with the corresponding geographical distances for peripheral (Mantel test; *r* = 0.11, *P* = 0.18) and central populations (Mantel test; *r* = 0.15, *P* = 0.23), respectively. The partitioning of total genetic variation of *O*. *rufipogon* among and within two groups of populations by AMOVA is shown in Table 5. Most genetic variation occurred among individuals within populations (66.54%) and among populations within groups (25.38%) rather than between two groups (8.08%). Across the six microsatellite loci, peripheral populations possessed a higher mean value (*F*_*IS*_ = 0.755) than central populations (*F*_*IS*_ = 0.673). However, the *F*_*IS*_ difference between peripheral and central populations was not significant (Table 2). The estimated *t* values apparently showed that the outcrossing rates might vary over the whole range of the species (Table 2). Peripheral populations tend to exhibit a decreased average outcrossing rate (central populations: *t* = 0.195; peripheral populations: *t* = 0.140). Nevertheless, these differences were not significant.

**Table 4:** Differences of *F*-statistics ^a^ between peripheral and central populations in *O*. *rufipogon* as detected by six microsatellite loci.

	Central populations			Peripheral populations		
Locus	*F*_*IS*_	*F*_*IT*_	*F*_*ST*_	*F*_*IS*_	*F*_*IT*_	*F*_*ST*_
**RM164** ^b^	0.66	0.874	0.642	0.734	0.849	0.414
	(0.102) ^d^	(0.042)	(0.111)	(0.083)	(0.078)	(0.219)
**RM241** ^b^	0.687	0.76	0.236	0.844	0.89	0.301
	(0.054)	(0.041)	(0.05)	(0.04)	(0.031)	(0.125)
**RM211** ^b^	0.558	0.637	0.18	0.742	0.76	0.063
	(0.053)	(0.04)	(0.048)	(0.112)	(0.111)	(0.031)
**RM253** ^b^	0.669	0.738	0.209	0.771	0.805	0.154
	(0.038)	(0.035)	(0.037)	(0.078)	(0.062)	(0.06)
**OSR28** ^b^	0.644	0.688	0.123	0.592	0.666	0.198
	(0.043)	(0.038)	(0.019)	(0.098)	(0.06)	(0.083)
**RM222** ^b^	0.836	0.871	0.216	0.812	0.851	0.217
	(0.067)	(0.052)	(0.027)	(0.086)	(0.066)	(0.125)
**Mean for each geographical group**	0.673*	0.760**	0.266***	0.755**	0.812*	0.233***
	**(0.041)**	**(0.039)**	**(0.075)**	**(0.037)**	**(0.033)**	**(0.044)**
***P*-value** ^c^	0.1835	0.4296	0.6461			
**95%CI**	**0.606**	**0.691**	**0.168**	**0.683**	**0.747**	**0.151**
	**(0.752)**	**(0.83)**	**(0.422)**	**(0.811)**	**(0.864)**	**(0.312)**
**Overall**	***F***_***IS***_ **= 0.691 (0.037); *F***_***IT***_ **= 0.778 (0.032); *F***_***ST***_ **= 0.284 (0.062)**					
**95%CI**	***F***_***IS***_ **= 0.63 (0.754); *F***_***IT***_ **= 0.723 (0.836); *F***_***ST***_ **= 0.201 (0.411)**					

^a^ Weir and Cockerham (1984) estimation of *F*_*IT*_ (*F*), *F*_*ST*_ (*θ*) and *F*_*IS*_ (*f*); all mean values for each locus were obtained after Jackknifing over populations. Statistically significant deviations from Hardy-Weinberg expectations are indicated by

* (*P* <0.05)

** (*P*<0.01) and

*** (*P*<0.001); overall estimates by bootstrapping over loci were also calculated, number of replicates = 999; nominal confidence interval = 95%

^b^ All values are given after Jackknifing over populations

^c^ The two-sided *P*-values obtained after 1,000 permutations

^d^ The standard deviations of the means are given in the parenthesis.

**Table 5:** Analysis of molecular variance (amova) to assess geographical partition between central and peripheral groups for 525 individual plants from 21 populations of *O*. *rufipogon* using six microsatellite loci.

Source of variation	d. f.	Sum of squares	Variance components	%Total	Φstatistics	*P*
**Among groups**	1	78.276	0.18578	8.08	Φ_CT_ = 0.08076	<0.0001
**Among populations within groups**	19	432.387	0.58393	25.38	Φ_SC_ = 0.27615	<0.0001
**Within populations**	751	1149.512	1.53064	66.54	Φ_ST_ = 0.33461	<0.0001
**Total**	771	1660.175	2.30036			

### Heterozygosity variation in seed and maternal generations between peripheral and central populations

For both two generations (i.e. seed and maternal samples), peripheral populations exhibited significantly lower levels of heterozygosities (seed populations: mean *H*_*O*_ = 0.0775, and *Hs* = 0.4567; maternal populations: mean *H*_*O*_ = 0.1488, and *Hs* = 0.5026) than central populations (seed populations: mean *H*_*O*_ = 0.2454, and *Hs* = 0.6885; maternal populations: *H*_*O*_ = 0.3652, and *Hs* = 0.6712) (seed populations: *H*_*O*_: *P* = 0.0003; *Hs*: *P* = 0.0002; maternal populations: *H*_*O*_: *P* = 0.0012; *Hs*: *P* = 0.0001). No significant difference was detected for the allelic richness (*R*_*S*_) between peripheral and central populations (seed populations: 2.2641 vs 2.4761; *P* > 0.05; maternal populations: 2.5344 vs 2.2966; *P* > 0.05). For the maternal samples, no significant difference was found for the *F*_*IS*_ values between peripheral and central populations. However, peripheral populations (mean *F*_*IS*_ = 0.8238) exhibited significantly higher *F*_*IS*_ values than those in central populations (mean *F*_*IS*_ = 0.6954) for the seed samples (*P* = 0.0281).

In both peripheral and central populations, maternal populations showed a significantly higher observed heterozygosity (*H*_*O*_) than seed populations (central populations: 0.3652 vs 0.2454, *P* = 0.0066; peripheral populations: 0.1488 vs 0.0775, *P* = 0.0203) (Table 6). In addition, significant difference was found between the observed and expected heterozygosity for both maternal and seed populations (*P* < 0.05) (data not shown). The *F*_*IS*_ values were significantly higher in seed populations than in maternal populations (*P* < 0.05) (Table 6).

**Table 6:** Comparisons of population genetic parameters between maternal and seed samples from peripheral and central populations of *O*. *rufipogon* as detected by six microsatellite loci ^a^.

Populations	*Sample Size*	*Rs*	*Ho* ^b^	*H*_*S*_	*F*_*IS*_ ^b^
**Central populations**					
GZ (maternal)	29	2.1976	0.3210***	0.6657	0.6493***
LD (maternal)	26	2.1706	0.3824***	0.6634	0.5936***
LX (maternal)	42	2.5216	0.3921***	0.6845	0.5887***
Pooling maternal populations	32	2.2966 (0.1953)	0.3652 (0.0386)	0.6712 (0.0116)	0.6105 (0.0337)
GZ (seed)	202	2.5599	0.2423***	0.6962	0.6982***
LD (seed)	241	2.3168	0.2571***	0.6746	0.6867***
LX (seed)	454	2.5517	0.2367***	0.6948	0.7013***
Pooling seed populations	299	2.4761 (0.1380)	0.2454 (0.0105)	0.6885 (0.0121)	0.6954 (0.0077)
*P*-value		0.2634	0.0066	0.1473	0.0131
**Peripheral populations**					
DX (maternal)	26	2.4790	0.1245***	0.4901	0.6463***
CL (maternal)	32	2.5497	0.1733***	0.5045	0.5671***
RP (maternal)	31	2.5746	0.1485***	0.5133	0.5676***
Pooling maternal populations	30	2.5344 (0.0496)	0.1488 (0.0244)	0.5026 (0.0117)	0.5937 (0.0456)
DX (seed)	458	2.1405	0.0589***	0.4232	0.8698***
CL (seed)	675	2.3197	0.0712***	0.4812	0.8529***
RP (seed)	352	2.3321	0.1023***	0.4656	0.7487***
Pooling seed populations	495	2.2641 (0.1072)	0.0775 (0.0224)	0.4567 (0.0300)	0.8238 (0.0656)
*P*-value		0.0166	0.0203	0.0689	0.0075

^a^ Allelic richness (*Rs*), the observed heterozygosity (*H*_*O*_), the gene diversity within sample (*H*_*S*_), and the heterozygote deficit within populations (*F*_*IS*_) for each studied population of *O*. *rufipogon* across six loci; mean values (*R*_*S*_, *H*_*O*_, *H*_*e*_ and *F*_*IS*_) are also given by pooling all central and peripheral populations, respectively

^b^ Statistically significant deviations from Hardy-Weinberg expectations are indicated by

*** (*P*<0.001).

## Discussion

### Decreased genetic variation towards the periphery of *O*. *rufipogon*

We obtained the evidence for decreased microsatellite variation at the periphery of the geographical range in *O*. *rufipogon*. Microsatellite analysis revealed significantly larger genetic variation in central populations than in peripheral populations. The rarefaction method confirmed significantly higher estimates of allelic richness for the central populations than those for the peripheral populations. The pattern is in agreement with theoretical predictions based on the assumption that drift or stable directional selection is major determinants of genetic diversity [1, 51]. Theory predicts that demographic instability inducing low effective population size, repeated bottlenecks or founder events, should lead to genetic impoverishment through the effect of accentuated drift. The most likely explanation for the reduction of genetic diversity in populations of *O*. *rufipogon* near the Northern limit of distribution is genetic drift that is apt to occur in severely isolated small peripheral populations. The Chinese almanac recorded that this wild rice species had a wider northern peripheral range (38° 5’ N) about 958–1,774 years ago [52] (Fig 1). As a species that is sensitive to photoperiod and temperature, *O*. *rufipogon* contracted its northern range southward and reached the present-day range (28° 14’ N) due to the gradual decrease of global temperature (and other related climatic changes) in the past centuries [52]. Peripheral populations of the species may have experienced multiple extinction events and severe reduction of population size, and thus the species exhibits the reduced population density and size in the present day [27]. Therefore, allelic variation has been lost and lower genetic diversity was observed in these isolated small peripheral populations than central populations as a result of natural adaptation.

It is also possible that the loss of genetic variation in the peripheral populations of *O*. *rufipogon* is due to the increased inbreeding within small populations. The positively high *F*_*IS*_ values with microsatellite analysis in the whole sample are indicative of an excess of homozygotes related to Hardy-Weinberg expectations and thus inbreeding. As pointed out by Brown [53], small population size and isolation by distance are of importance to enhance inbreeding and exhibit a deficiency of heterozygotes. In addition to the observation that peripheral populations exhibited significantly higher *F*_*IS*_ values than those in central populations, we found that a positive correlation between population size and microsatellite diversity was significant in *O*. *rufipogon*. The result is expected based on the population genetic theory, which predicts that genetic variation should increase with effective population size [54], thus further supporting the previous empirical data [5, 12, 55]. Therefore, larger inbreeding due to smaller effective population size might explain the decreased genetic variation observed in smaller populations at the margins of the geographical range.

Reduced gene flow promotes inbreeding and genetic drift and thus results in the decreased genetic variation in the peripheral populations. We found the significantly positive correlation between levels of microsatellite diversity and the distance from the northern margin and northeastern margin in *O*. *rufipogon*. The finding suggests that gene flow from central to peripheral populations of *O*. *rufipogon* may be reduced in proportion to their extent of geographical distance. It is understandable that historical contraction of geographical range in *O*. *rufipogon* might have led to directional gene flow from the central to peripheral populations. Reduced gene flow, according to Mayre's [56] statement, may be used to explain gradual decline of genetic diversity toward the extreme northern margin. Populations located at range margins are more distant from the sources of immigrants and are thus more subject to genetic bottlenecks that eventually lead to the reduction of genetic diversity [33, 57–59].

### Increased genetic divergence of peripheral populations from central populations

Although some researchers reported different genetic structure of peripheral populations from central populations [13, 16, 60], in the present study, we failed to detect such a significantly different distribution of genetic variation within *O*. *rufipogon*. Nevertheless, genetic drift in small peripheral populations could be important to have promoted the likelihood of its genetic divergence from central populations of *O*. *rufipogon*. It could at least cause peripheral populations to have different alleles and/or allele frequencies than central populations as detected by microsatellite loci in this study (data not shown but available upon request). Studies of allele frequencies using enzyme electrophoresis and microsatellite analysis have also detected such differences in many other plant species [12, 55, 60, 61], which may potentially indicate the increased differentiation of peripheral populations.

Mating system is important to determine patterns of intraspecific variation in gene diversity [62], and could partially influence the *F*_*ST*_ statistic [63, 64]. Estimates of outcrossing rate showed that peripheral populations had a decreased average outcrossing rate in comparison to central populations without significant difference. Compared with outbreeding plants, inbreeding species showed lower levels of genetic diversity but a markedly greater variation among populations [62]. Therefore, our finding that peripheral populations tend to exhibit an increased inbreeding could also explain a lowered genetic diversity within but increased genetic divergence among peripheral populations in comparisons to the central populations of *O*. *rufipogon*.

Natural selection, in addition to genetic drift, may serve as another primary force determining the increased genetic divergence of peripheral populations from central populations [5]. As discussed above in *O*. *rufipogon*, ecologically peripheral populations are mostly rather small and inbred, thus “homoselection” due to limited ecological niches; central populations, however, are dominated by “heteroselection” due to the most favorable environmental conditions [65]. Moreover, environmental favorableness presumably decreases with the increased distance from the center [66]. Considering the data presented in our previous study on ecological differentiation among natural populations of *O*. *rufipogon* [67], for example, there was an observation, which may be relevant to demonstrating natural selection that enhances genetic divergence of peripheral populations. First, with the increased latitudes the proportion of asexual tends to decrease and that of sexual reproduction increases as evidenced by larger 100-seeds weight and increased rate of seed-setting in peripheral populations; and second, as latitudes increase natural populations showed more sensitive to its photoperiod as indicated by that the increased daylight length makes the initial heading stage earlier in peripheral populations. Directional selection may be responsible for such a gradual divergence from central to peripheral populations of *O*. *rufipogon* under environmental changes in a clinal manner. In order to prove natural adaptation is not the only reason for the lowered genetic diversity in peripheral populations, we compare the genetic diversities between peripheral populations (RP, ZP) (Rs = 2.721, HO = 0.1233, and Hs = 0.5571) and central populations (DY, LX, LA, TK, KE, ZC, BL, NB) (Rs = 3.559, HO = 0.3043, and Hs = 0.7006) which almost distributed in the same latitude (23°–24° 2’ N). The results suggest that the lowered genetic diversity in peripheral populations even if the similar natural environments and climates in populations. Further evidence should be sought to test whether most observed gene variation is of adaptive significance and thus is maintained by some form of balancing selection.

### Inbreeding depression occurred within peripheral and central populations

The variation of homozygote frequency between seed populations and maternal or adult populations may be a better parameter to detect inbreeding depression. For both peripheral and central populations of *O*. *rufipogon*, we detected significantly low observed heterozygosity (*H*_*O*_) and high heterozygote deficit within populations (*F*_*IS*_) values in seed samples in comparisons with maternal samples, indicating an excess of homozygotes. The increased frequencies of homozygotes in seed populations imply that a great number of hetorozygotes had been eliminated during the transformation from adult plants to seeds in *O*. *rufipogon*. When an individual self-fertilizes or mates with a relative, the lethal or highly deleterious recessive alleles are often made homozygous, and thus an inbreeding depression occurs within seed populations of such an outcrossing species. The observation may come from inbreeding and thus excessive homozygotes as a result of the rapidly decreased population sizes in the wild rice species [29].

Our data further suggest that, comparing with central populations, a serious inbreeding depression might occur within peripheral populations of *O*. *rufipogon*. There is no significant difference of the *F*_*IS*_ values between peripheral and central populations for maternal samples. However, for seed samples, significantly lower observed heterozygosity (*H*_*O*_) and higher *F*_*IS*_ values were found in peripheral populations than those in central populations. The results indicate an excess of homozygotes and thus high inbreeding depression in peripheral populations. It is likely that small effective population size promotes mating events among relatives within a peripheral population. As a result, more hetorozygotes had been removed in peripheral populations of *O*. *rufipogon* than those in central populations when transforming from adult plants to seeds.

### Conservation implications of peripheral populations of *O*. *rufipogon*

Knowledge of levels and partitioning of genetic variability within and among peripheral and central populations obtained here suggest that northern and northeastern peripheral populations of *O*. *rufipogon* represent a significant proportion of the total genetic variation of the species. As a consequence, these populations should be considered in the maintenance of genetic diversity in the species. A reduction in genetic diversity is considered fatal for the future survival of a species and its abilities to adapt to changing environments [68–70]. This has led to the rational that conservation plans should mainly focus on protecting most of diverse and large central populations. For reasons discussed below, however, this is not necessarily a reasonable strategy. First, due to less favorable habitats at the margin of a species’ distribution area, the selection regimes in such peripheral populations appear usually often more harsh and divergent. Considering well-adapted “stress” genotypes in the peripheral populations, they might possess higher value to conservation than central populations, although the overall levels of genetic diversity are lower than in the central populations [71]. Second, the fitness of a population is not necessarily dependent on, or correlated with, microsatellite variability that has been characterized. Speciation may be more likely to occur in peripheral populations because of the pronounced genetic differentiation and limited gene flow [72, 73]. Thus, peripheral populations of *O*. *rufipogon* such as those studied here, may be valuable in the evolutionary process and should be taken into consideration with respect to the conservation. Third, our results indicate an excess of homozygotes and thus high inbreeding depression due to lowered effective population size in peripheral populations of *O*. *rufipogon*. The evidence that heterozygote advantage might be more pronounced in peripheral populations [72] results in the maintenance of a higher heterozygosity at some loci than might be expected on basis of reduced population size. Therefore, the potential for loss of genetic variation and potential fitness effects as a result of inbreeding should be seriously considered in planning for the conservation and management of the species.

For protecting the integrity of the whole gene pool of *O*. *rufipogon*, patterns of genetic variation and population structure have substantial implications for gene conservation strategies. Comparing with continuous central populations, northern and northeastern peripheral populations possess lowered genetic diversity but increased population structure. This finding is further supported by a recent genome-wide study that documented significant correlations between levels of genomic diversity and geographic distributions in Chinese *O*. *rufipogon* populations [74]. The striking differences have implications for relatively different conservation strategies for either germplasm sampling of *ex situ* conservation or setting *in situ* reserves. Comparatively speaking, sampling strategy for gene conservation purposes will be relatively more critical for peripheral populations than in central populations. Conserving genetic diversity in northern peripheral populations may require larger reserves for *in situ* conservation than required in central populations.

## Conclusions

Historical extinction events, demographic changes, and environmental conditions near the northern and northeastern margins of *O*. *rufipogon* favor inbreeding and possibly selfing. Genetic drift, reduced gene flow, and possible local selection, consequently lead to lowered gene diversity, accelerated genetic divergence and increased inbreeding depression in peripheral populations of *O*. *rufipogon*. These characteristics make these peripheral populations likely to develop local adaptations to extreme environmental changes, and therefore are possible to be of great interest for wild rice germplasm conservation. To meet needs of the future global environmental changes further studies are of potential importance to guide rice breeder to find novel alleles for the rice improvement programs.
